# Supplementary material for: Prolonged Aggressive Experience Accelerates Resolution of Inflammation in Blood and Microglia After Repeated LPS Treatment
Source: Int J Mol Sci. 2025 Dec 13;26(24):12007. doi: 10.3390/ijms262412007 (PMC12733220; doi:10.3390/ijms262412007)
Supplement: Supplementary file 1 [file ijms-26-12007-s001.zip › supplementary tables.pdf]

Table S1. Results of factorial analysis of behavioral tests.

Test	Parameters		"Aggression" effect	"Cluster" effect	"Treatment" effect	Time point effect	"Cluster""treatment" effect
**Pathological aggression test**	Attack latency	P1	ns	F(1,43)=112.1, p<0.001	×	F(2,86)=7.9, p<0.001	×
		P2	ns	F(1,43)=62.65, p<0.001	×		×
		P3	F(2,43)=5.9, p=0.02	F(2,43)=28.68, p<0.001	ns		ns
	Attack time	P1	ns	F(1,43)=14.63, p<0.001	×	F(2,86)=10.32, p<0.001	×
		P2	ns	F(1,43)=15.32, p<0.001	×		×
		P3	ns	[F(2,43)=10.45, p<0.001]	ns		ns
	Locomotor activity	P1	ns	F(1,43)=8.1, p<0.001	×	F(2,86)=26,9, p<0.001	×
		P2	ns	F(1,43)=5.9, p=0.005	×		×
		P3	F(2,43)=27.7, p<0.001	F(2,43)=16.2, p<0.001	F(2,43)=9.9, p=0.002		ns
**Aggressive confrontation test to a moving partner**	Attack latency	P1	×	F(1,43)=71.29, p<0.001	×	F(2,86)=8.98, p=0.004	×
		P2	×	F(1,43)=243.04, p<0.001	×		×
	Attack time	P1	×	F(1,43)=7.18, p=0.002	×	F(2,86)=20.533, p<0.001	×
		P2	×	F(1,43)=13.17, p<0.001	×		×
**Partition test**	Number of approaches		×	ns	×	×	×
	Time near partition		×	F(1,43)=3.65, p=0.034	×	×	×
**Elevated plus maze**	Time in open arm		F(1,26)=16.03; p<0.001	F(1,26)=5.97; p=0.003	×	×	×

	Time in closed arm	F(1,26)=5.096, p=0.03	ns	×	×	×
	Time in the center	F(1,26)=10.79; p=0.002	F(1,26)=5.10; p=0.007	×	×	×
	Total number of entrances	ns	ns	×	×	×
	Poking number	F(1,26)=8.21; p=0.008	F(1,26)=5.14; p=0.006	×	×	×
**Light-dark box**	Latent time	ns	ns	F(2,27)=7.567; p=0.010	×	ns
	Time in light box	ns	ns	ns	×	ns
	Number of entrances	ns	ns	F(2,27)=14.100; p<0.001	×	ns
	Poking number	F(2,27)=93.9; p<0.001	F(2,27)=33.4; p<0.001	ns	×	ns

Notes: ns - analysis did not find any significant difference, × - analysis was not conducted due to differences in included factors for different behavioral tests.

Table S2. Results of factorial analysis of gene expression.

System	*Gene*		"Aggression" effect	"Treatment" effect	"Aggression""treatment" effect
**Inflammation**	*Aif1*	Hypothalamus	ns	F(1,54)=66, p<0.001	F(3,54)=7.8, p<0.008
		NAc	ns	ns	ns
	Cxcl10	Hypothalamus	F(1,53)=5.1, p<0.03	F(1,53)=8.0. p<0.007	F(3, 53) = 16.1, p<0.001
		NAc	ns	F(1,48)=8.1, p<0.007	F(1,48)=4.4, p<0.05
	Il-1β	Hypothalamus	ns	F(1,53)=45, p<0.001	ns
		NAc	ns	F(1,48)=14.4, p<0.001	ns
	Trem2	Hypothalamus	F(1,54)=5.2, p<0.03	F(1,54)=13.4, p<0.001	ns
		NAc	F(1,48)=11.1, p<0.002	ns	ns
	Gfap	Hypothalamus	F(1,54)=4.9, p<0.04	ns	ns
		NAc	F(1,48)=4.9, p<0.03	F(1,48)=8.8, p<0.005	ns
	Mrc1	Hypothalamus	ns	F(1,54)=5.9, p<0.02	ns
	Traf6	Hypothalamus	ns	ns	F(3,54)=5.6, p<0.03
**HPA**	*Fkbp5*	Hypothalamus	ns	ns	ns
		NAc	ns	ns	ns
	Nr3c1	Hypothalamus	ns	ns	ns
		NAc	F(1,48)=62.5, p<0.001	ns	ns
	Crh	Hypothalamus	ns	ns	ns
	Crhr1	Hypothalamus	ns	ns	ns
**Dopamine**	*Drd1*	Hypothalamus	F(1,54)=4.8, p<0.04	ns	ns

	NAc	F(1,48)=5.2, p<0.03	ns	ns
Drd2	Hypothalamus	ns	ns	ns
	NAc	ns	ns	ns
Drd3	Hypothalamus	ns	ns	ns
	NAc	ns	ns	ns
Ppp1r1b	Hypothalamus	ns	ns	ns
Slc6a3	Hypothalamus	ns	ns	ns
Maoa	Hypothalamus	ns	ns	ns
Th	Hypothalamus	ns	ns	ns

Notes: ns - analysis did not find any significant difference, × - analysis was not conducted due to differences in included factors for different behavioral tests.

Table S3. Primer sequences used for quantitative real-time PCR

Primer sequences used for quantitative real-time PCR with fluorescent probes		
**Gene**	**Sequence 5’→3’**	
Abcb1a	For	CGATAAAAGAGCCATGTTTGC
	Rev	CTGATCTTGTGTATCTGTCTTCC
	Probe	ROX-TGCTGGTGTGCTCATAGTTGCCT-BHQ2
Aif1	For	GCTTTTGGACTGCTGAAGGC
	Rev	GAAGGCTTCAAGTTTGGACG
	Probe	ROX-AGAGAGGCTGGAGGGGATC-BHQ2
Crh	For	GGAGAAGAGAGCGCCCCTAA
	Rev	AAGAAATTCAAGGGCTGCGG
	Probe	ROX-ATGCTGCTGGTGGCTCTGTCGTCC-BHQ2
Crhbp	For	TCCATACCAGCACCAAAACTG
	Rev	CAGCTCCACAAAGTCACCAG
	Probe	ROX-ACTGAAGGCCATGCAAGTGTCCGA-BHQ2
Crhr1	For	GGTCCTGCTGATCAACTTTA
	Rev	ACATGTAGGTGATGCCCA
	Probe	ROX-CTGGGGTGCCTTTCCC-BHQ2
Cxcl10	For	GAATGAGGGCCATAGGGAA
	Rev	CATCGTGGCAATGATCTCAA
	Probe	ROX-CCCTGCGAGCCTATCCTGCCCACGT-BHQ2
Drd1	For	GGAGAGCAGAGCGAGCG
	Rev	GGTTCAGTGCTCCAGGTCG
	Probe	ROX-AGCGTGGTCTCCCAGATC-BHQ2
Drd2	For	ROX-CGCTGTGGCCGATCTTCTGGTGGCC-BHQ2
	Rev	CAACTACTATGCCATGCTGC

	Probe	ATGTCACAGTGAATCCTGCT
Drd3	For	ROX-AGGGCAGGACACAGCAAAGG-BHQ2
	Rev	CCATCAGCATAGACAGGTAC
	Probe	CAAAGGGAACATAGAAGGAC
Fkbp5	For	AGAATCAAACGGAAAGGCGAG
	Rev	CTCGGCAATCAAATGTCCTTC
	Probe	ROX-CCAAACGAAGGAGCAACGG-BHQ2
Gfap	For	CCTGAGAGAGATTCGCACTC
	Rev	GACTCCAGATCGCAGGTCAAG
	Probe	ROX-GCAAGAGACAGAGGAGTGG-(BHQ-2)
Hk1	For	ACATTGTCTCCTGCATCTCC
	Rev	GCTTTGAATCCCTTTGTCCAC
	Probe	Cy5-CCTTCTCGTTTCCCTGCAAG-BHQ2
Il-1β	For	CCTGTTCTTTGAAGTTGACGG
	Rev	CTGAAGCTCTTGTTGATGTGC
	Probe	ROX-CTGCTTCCAAACCTTTGACCTGG-BHQ2
Mrc1	For	TGTAAGATGGTATCACAAAGC
	Rev	TGAAGGTGGATAGAGTGG
	Probe	CGGAGGGTGCAGACAAAGGCT
Nr3c1	For	ATGTATGACCAATGTAAACACA
	Rev	GCTCTTCAGACCTTCCTTAG
	Probe	ROX-TGCAGGTATCCTATGAAGAG-BHQ2
Pik3c3	For	GGATTGGCTGGACAGATT
	Rev	CTCCTTGTCATCGCACTT
	Probe	HEX-ACTTGATGGTTGAGTTTCGCTGTGT-BHQ1
Traf6	For	CTACGATGTGGAGTTTGACC
	Rev	ATTTCATTGTCAACTGGGCA

	Probe	ROX-GTGGCCACAGGTTCTGCAAAGCCTGCA-BHQ2
Trem2	For	CGTCACCATCACTCTGAA
	Rev	CCAGCATCTTGGTCATCTA
	Probe	AGAGGCTGAGGTCCTGCAGAAAGT
Itgam	For	AAATTCGGTGATCCCTTG
	Rev	CTTAGATGCGATGGTGTC
	Probe	TCTCTGCGGGACTGTGGTTTGTT
**Primer sequences used for fluorescence quantitative real-time PCR with an intercalating dye**		
**Gene**	**Primer Sequences**	**Sequence 5'→3'**
Maoa	For	AATGAGGATGTTAAATGGGTAGATGTTGGT
	Rev	CTTGACATATTCAACTAGACGCTC
Ppp1r1b	For	CCACCCAAAGTCGAAGAGAC
	Rev	GCTAATGGTCTGCAGGTGCT
Slc6a3	For	TTCACTGTCATCCTCATCTCTTTC
	Rev	TCAAAATACTCAGCAGCGGGTG
Th	For	CTGTGGCTACCGAGAGGA
	Rev	GGAGAACTGGGCAAATGT
