# Supplementary material for: Prolonged Aggressive Experience Accelerates Resolution of Inflammation in Blood and Microglia After Repeated LPS Treatment
Source: Int J Mol Sci. 2025 Dec 13;26(24):12007. doi: 10.3390/ijms262412007 (PMC12733220; doi:10.3390/ijms262412007)
Supplement: Supplementary file 1 [file ijms-26-12007-s001.zip › Supplementary Figures.pdf]

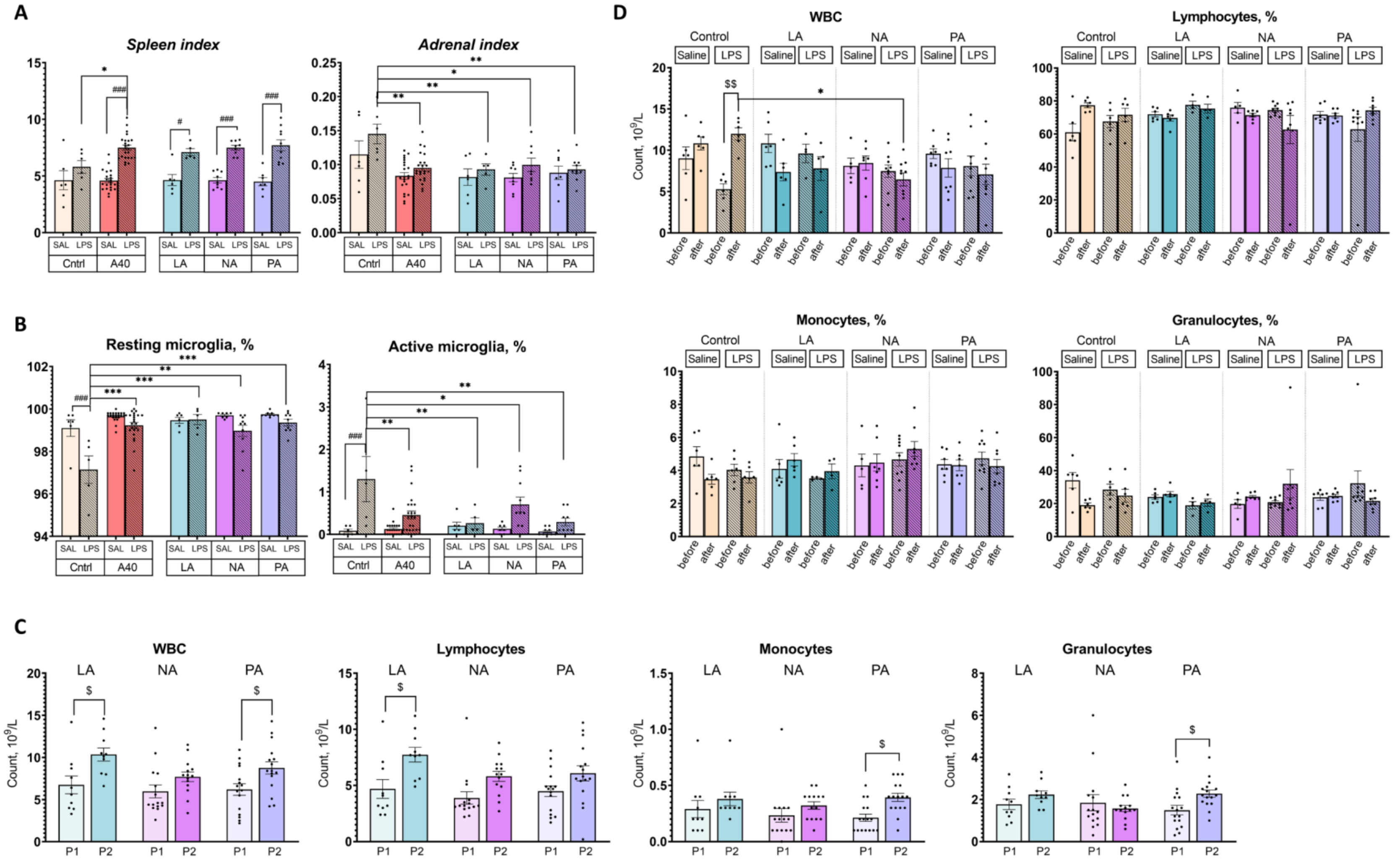

Figure S1. Response to LPS treatment in different groups of aggressors. A. Organ indexes (organ weight (g)/body weight (g)); B. Changes in microglial population 5 days after LPS treatment. C. Complete blood count changes in mice before (P1) and after prolonged experience of aggression (P2); D. Complete blood count changes after 7-day LPS treatment; E. Gating strategy of microglial cells; Control (Cntr) - mice without aggressive experience; LA - Low Aggression mice; NA - Non-pathological Aggressors; PA - Pathological Aggressors; Saline - 7-day saline treatment; LPS - 7-day LPS treatment; P1 - beginning of aggressive confrontations; P2 - the end of aggressive confrontations; before - CBC before LPS/Saline treatment; after - CBC after LPS/Saline treatment; * $p < 0.05$, ** $p < 0.01$, *** $p < 0.001$ Aggressors vs Control; # $p < 0.05$, ## $p < 0.01$, ### $p < 0.001$ LPS vs Saline, two-way ANOVA with Tukey HSD post-hoc test; $$ $p < 0.01$, $$$ $p < 0.001$ between time points (P1 vs P2), repeated measures ANOVA with Tukey HSD post-hoc test.

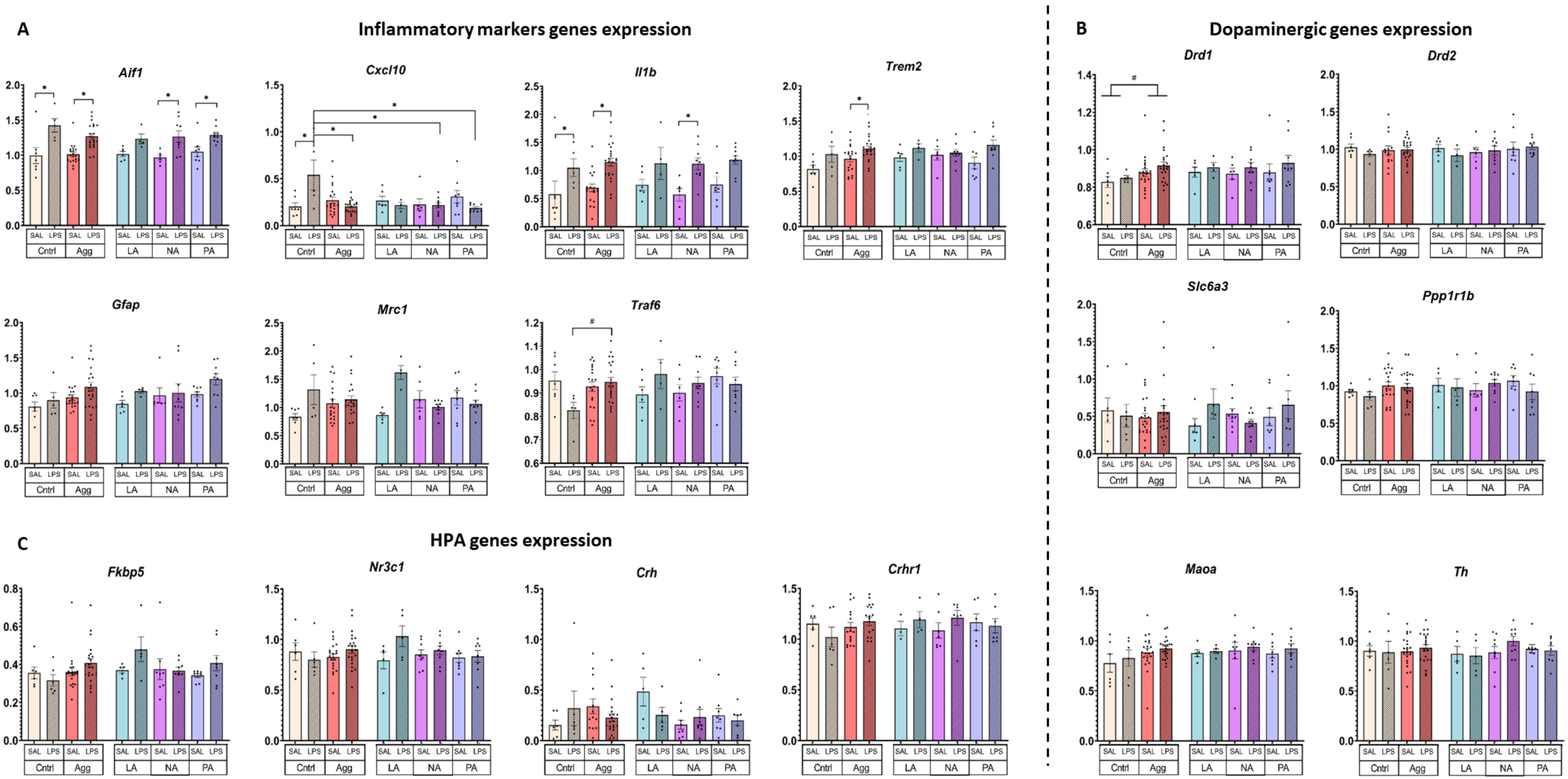

Figure S2. Gene expression in the hypothalamus. A - Inflammatory markers genes expression; B - dopaminergic genes expression; C - HPA genes expression. Control - mice without aggressive experience Aggressors—mice with 30-day aggressive experience, LA—Low Aggression mice, NA—Non-pathological Aggressors, PA—Pathological Aggressors, Saline - 5-day saline treatment, LPS - 5-day LPS treatment. Y-Axis—expressions normalized on reference genes. $p < 0.05$, $p < 0.01$ LPS vs Saline in group. # $p < 0.05$ Aggressors vs Control, two-way ANOVA with Tukey HSD post-hoc test.

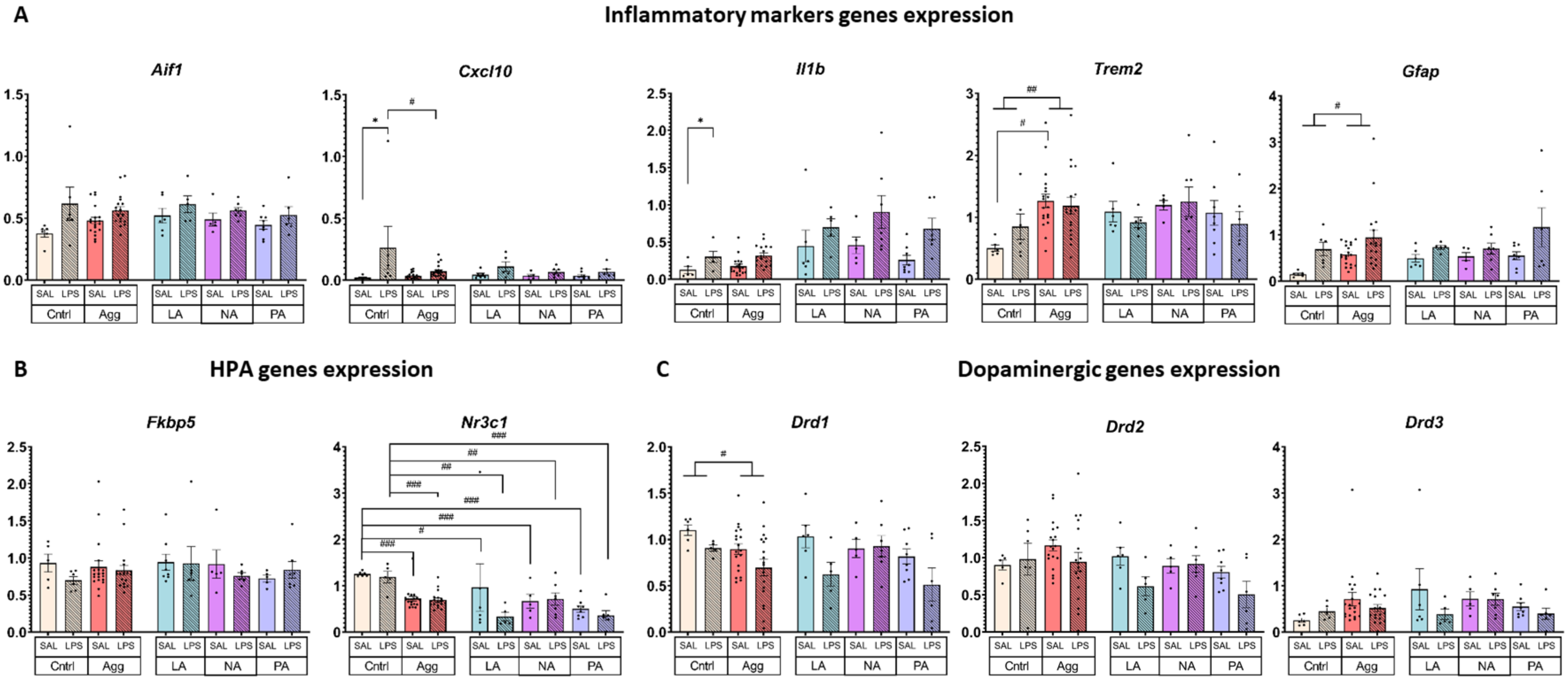

Figure S3. Gene expression in the nucleus accumbens. A - Inflammatory markers genes expression; B - HPA genes expression; C - dopaminergic genes expression. Control—mice without aggressive experience Aggressors—mice with 30-day aggressive experience; LA—Low Aggression mice; NA—Non-pathological Aggressors; PA—Pathological Aggressors. Saline - 5-day saline treatment, LPS - 5-day LPS treatment. Y-Axis—expressions normalized on reference genes. $p < 0.05$, $p < 0.01$ LPS vs Saline in group. # $p < 0.05$ Aggressors vs Control, two-way ANOVA with Tukey HSD post-hoc test.
